# Aptitude for Research Among Medical Students in India: Identifying the Challenges

**DOI:** 10.7759/cureus.68885

**Published:** 2024-09-07

**Authors:** Venkata Sai Abhilash Meda, Arshia Firdaus, Anju Pradeep, Harshaman Kaur, Nihar Patel, Advait M Vasavada

**Affiliations:** 1 Internal Medicine, Jawaharlal Institute of Postgraduate Medical Education and Research, Puducherry, IND; 2 Internal Medicine, Deccan College of Medical Sciences, Hyderabad, IND; 3 Internal Medicine, Private Practice, Kollam, IND; 4 Internal Medicine, Sri Guru Ram Das Institute of Medical Sciences and Research, Amritsar, IND; 5 Internal Medicine, B J Medical College, Ahmedabad, IND; 6 Family Medicine, University of Nebraska Medical Center, Omaha, USA; 7 Internal Medicine, M P Shah Medical College, Jamnagar, Jamnagar, IND; 8 Family Medicine, California Institute of Behavioral Neurosciences & Psychology, Fairfield, USA

**Keywords:** aptitude, challenges, medical research, medical sciences, undergraduate research

## Abstract

Introduction

Understanding the reasons behind the undervaluation of research among undergraduate medical students in India is crucial for advancing medical knowledge. This cross-sectional study aims to investigate the knowledge, interest, and barriers faced by 606 Indian medical students through an online questionnaire distributed via social media platforms.

Aims

To identify the knowledge of research and the challenges faced by medical students to pursue research in their undergraduate education.

Methods and material

This was a prospective observational study carried out over a period of one month in October 2022. Study approval was taken from the Genebandhu Independent Ethics Committee (Reference Number- ECG004/2022). The responses obtained from the questionnaire were recorded in Google Sheets and transferred to Microsoft Excel (Redmond, WA, USA).

Results

Among the participants, 46.53% were male and 53.47% were female, primarily from the first (11.39%) and second (9.90%) years of their medical education. Notably, the majority had undertaken United States Medical Licensing Examination (USMLE) (54.95%), followed by National Eligibility cum Entrance Test (Postgraduate) (NEET-PG) (34.65%) and Professional and Linguistic Assessments Board (PLAB) (7.92%). The study revealed that 78.22% considered research pivotal to their academic trajectory, with a mean age of 23.14 years (SD=2.82) and an average research knowledge score of 2.75 (SD=1.31). The type of postgraduate exam significantly influenced enthusiasm for research activities (p<0.001), with higher enthusiasm among those preparing for exams abroad. Nonetheless, the type of medical college did not significantly affect interest in research activities (p=0.4879).

Conclusion

Addressing the undervaluation of research among undergraduate medical students in India is imperative. The curriculum should integrate robust support mechanisms to nurture research skills, emphasizing its importance for future medical practitioners. This could involve seminars, courses, and interactive sessions aimed at fostering research acumen among students.

## Introduction

Research is an essential part of the advancing of medical knowledge. It contributes to the development of new medicines, procedures, and tools, that can help in improvement of diagnosis and treatment [1,2]. The advancement of clinical medicine depends on the discoveries that take place through research. However, its incorporation into the undergraduate medical curriculum widely varies among the countries.

It has been observed that the number of physician-scientists has been declining over time [3,4], urging this trend be counteracted. As a result, evaluating medical students’ attitudes towards research is crucial in understanding the obstacles faced in order to better overcome them and promote research for future generations of healthcare professionals. Encouraging research among medical students would also promote the view of research as a possible career choice among medical graduates [5,6].

It is known that medical research is valued for positions in postgraduate courses in countries like the United States and the United Kingdom and is often incorporated into the medical curriculums there [7]. In the Indian medical system, however, for a Bachelor of Medicine, Bachelor of Surgery (MBBS) degree, there is no compulsory incorporation of research [8].

Hence, ultimately understanding the current aptitude for research among medical students of India along with the challenges faced by them is of prominence. Not only can it enhance the educational aspects of the existing medical curriculum, but it also holds the potential for significant contributions to the medical community.

The aim of this study is to identify the aptitude for research and the challenges faced by medical students to pursue research in their undergraduate education.

## Materials and methods

A cross-sectional type of observational study was conducted virtually over a six-month period, from October 2022 to April 2023. Ethics Committee Approval was obtained prior to the start of the study (GeneBandhu Ethics Committee Protocol Number ECG004/2022, dated October 5, 2022).

For this study, a questionnaire was made to collect details like demographic characteristics, research knowledge, interest, participation, and challenges encountered in research activities, utilizing a 5-point Likert scale for quantitative analysis. A pilot study was conducted with five participants for testing, and this was not included in the main study. This pre-tested questionnaire was converted into Google Forms and circulated to the target participants using social media platforms like WhatsApp, Facebook, and Instagram.

Participants who were medical students, interns or medical graduates from India were included into the study. Participants from countries other than India were excluded from the study.

The data was recorded in Google Sheets using Google Forms. The data was transferred to Microsoft Excel (Redmond, WA, USA) for further analysis. Statistical analysis was done using Microsoft Excel's Function tool.

## Results

A total of 606 participants were included in the study, with 46.53% males and 53.47% females. The distribution of participants across different academic years showed that the majority were in the first year (11.39%) and second year (9.90%) of their medical education.

Regarding the participants' background, 45.54% were graduates with less than two years of experience, and an almost equal number of students belonged to government (50.00%) and private (42.08%) medical colleges. A small percentage (7.92%) of participants had studied outside India. In terms of the exams taken by the participants, the majority had taken the United States Medical Licensing Examination (USMLE) (54.95%), followed by the National Eligibility cum Entrance Test (Postgraduate) (NEET-PG) (34.65%) and the Professional and Linguistic Assessments Board (PLAB) (7.92%).

The study found that 78.22% of the participants considered research an important asset to their graduate training path. The mean age of the participants was 23.14 years, with a standard deviation of 2.82. The participants' overall knowledge about research, assessed through five questions, had a mean score of 2.75 (SD=1.31).

Analyzing the gap in research knowledge between government and private medical colleges, no significant difference was observed (p=0.78). Similarly, there was no significant difference in research knowledge between students writing different exams (p=0.4299). These findings suggest that the type of medical college or the exam taken does not significantly affect research knowledge.

Examining the participants' aptitude for research, the study found that the majority (57.92%) were interested in conducting medical research, while only 25.25% had participated in conducting research. Additionally, 7.43% had published a case report/case series, 7.43% had published a research original article, and 6.44% had published a review article. A smaller proportion (17.82%) had participated in presenting a poster/oral paper at a conference, and 16.34% had participated in the Indian Council of Medical Research-Short Term Studentship (ICMR-STS) program.

Further analysis explored the determinants of enthusiasm in research activities. It was observed that the type of postgraduate exam significantly influenced enthusiasm for research activities (p<0.001), with a higher percentage of participants preparing for exams outside India expressing enthusiasm compared to those preparing for Indian exams. However, the type of medical college (private or government) did not significantly impact the participants' interest in research activities (p=0.4879).

Regarding the availability of support for research activities, medical school encouragement was reported by 49.51% of participants, while 81.6% of private college students and 81.5% of government college students received support. MBBS batchmates, seniors, professors/faculty, and online research platforms were also identified as sources of support. Table 1 provides characteristics of study participants

**Table 1:** Characteristics of study participants. Mean age of participants: 23.14 years with an SD of 2.82 Mean of correctly answered questions in assessment of knowledge about research by participants (out of five questions): 2.75 (SD- 1.31) USMLE: United States Medical Licensing Examination, NEET-PG: National Eligibility cum Entrance Test (Postgraduate), PLAB: Professional and Linguistic Assessments Board, INICET: Institute of National Importance Combined Entrance Test

Sr. No.	Characteristic criteria	Number of Students (Out of 606)		Percentage (%)
1.	Male	282		46.53
2.	Female	324		53.47
3.	1st Year	69		11.39
4.	2^nd^ Year	60		9.90
5.	3^rd^ Year	66		10.89
6.	4^th^ Year	57		9.41
7.	Intern Year	78		12.87
8.	Graduate (< 2 years)	276		45.54
9.	Government	303		50.00
10.	Private	255		42.08
11.	Outside India	48		7.92
12.	NEET-PG	210		34.65
13.	USMLE	333		54.95
14.	PLAB	48		7.92
15.	INICET	9		1.49
16.	Other	6		0.99
17.	Is Research an important asset to your graduate training path	YES -	474	78.22
		NO-	132	21.78

Table 2 shows the assessment of gap of research knowledge between government and private medical colleges. Table 3 shows the assessment of gap of research knowledge among students writing different exams. Table 4 depicts aptitude of research. Table 5 shows the participation in research activities. Table 6 shows the determinants of enthusiasm in research activities in medical colleges. Table 7 depicts the determinants of enthusiasm in research activities in medical colleges.

**Table 2:** Assessment of gap of research knowledge between government and private medical colleges. Inference: No difference in knowledge between government and private medical college students.

Sr. No.	Correct answers	Private colleges	Government colleges	p value
		n=255	n=303	
1.	Mean	2.72	2.88	0.78
2.	SD	1.34	1.31	Not significant

**Table 3:** Assessment of gap of research knowledge among students writing different exams. *Indian exams: NEET, INICET **Outside exams: USMLE, PLAB, others Inference: No difference in knowledge of research among the two groups USMLE: United States Medical Licensing Examination, NEET: National Eligibility cum Entrance Test, PLAB: Professional and Linguistic Assessments Board, INICET: Institute of National Importance Combined Entrance Test

S No.	Correct answers	Indian exams*	Outside exams**	p value
		n=219	n=381	
1.	Mean	2.74	2.76	0.4299
2.	SD	1.35	1.31	Not significant

**Table 4:** Aptitude of research MBBS: Bachelor of Medicine, Bachelor of Surgery, ICMR-STS: Indian Council of Medical Research-Short Term Studentship

Sr. No.	Aptitude criteria	Answers	Number (Out of 606)	Percentage (%)
1.	When did you first hear about medical research?	1^st^ year MBBS	240	39.60
		2^nd^ year MBBS	138	22.77
		3^rd^ year MBBS	84	13.8
		4^th^ year MBBS	51	8.42
		Internship	54	8.91
		After graduation	39	6.44
2.	Where did you hear about medical research?	MBBS Batchmates	150	24.75
		MBBS Seniors	105	17.33
		MBBS students at other colleges	30	4.95
		Faculty/Teachers	183	30.20
		Family	24	3.96
		Social media	99	16.34
		Other friends	6	0.99
		Others	9	1.49
3.	Knowledge of ICMR-STS	Yes	468	77.23
		No	138	22.77
4.	When did you first hear about ICMR-STS?	1^st ^year MBBS	168	27.72
		2^nd^ year MBBS	153	25.25
		3^rd^ year MBBS	51	8.42
		4^th^ year MBS	36	5.94
		Internship	51	8.42
		N/A	147	24.26

**Table 5:** Participation in research activities. ICMR-STS: Indian Council of Medical Research-Short Term Studentship

Sr. No.	Category	Answer	Number (Out of 606)	Percentage (%)
1.	Interested in conducting medical research	Strongly Agree	351	57.92
		Agree	150	24.75
		Neutral	87	14.36
		Disagree	9	1.49
		Strongly Disagree	9	1.49
2.	Participated in conducting medical research.	Yes	153	25.25
		No	453	74.75
3.	Interested in publication of a case report/case series	Strongly Agree	363	59.90
		Agree	150	24.75
		Neutral	84	13.86
		Disagree	3	0.50
		Strongly Disagree	6	0.99
4.	Published a case report/case series	Yes	45	7.43
		No	561	92.57
5.	Interested in publication of a research - original article	Strongly Agree	357	58.91
		Agree	171	28.22
		Neutral	66	10.89
		Disagree	6	0.99
		Strongly Disagree	6	0.99
6.	Published a research- original article	Yes	45	7.43
		No	561	92.57
7.	Interested in publication of a review article	Strongly Agree	309	50.99
		Agree	186	30.69
		Neutral	99	16.34
		Disagree	6	0.99
		Strongly Disagree	6	0.99
8.	Published a review article	Yes	39	6.44
		No	567	93.56
9.	Interested in presentation of poster/oral paper at a conference	Strongly Agree	321	52.97
		Agree	174	28.71
		Neutral	90	14.85
		Disagree	15	2.48
		Strongly Disagree	6	0.99
10.	Participated in presentation of poster/oral paper at a conference.	Yes	108	17.82
		No	450	74.26
		Unanswered	48	7.92
11.	Participated in ICMR-STS	Yes	99	16.34
		No	507	83.66
12.	Completed ICMR-STS study in the proposed timeframe?	Yes	39	6.44
		No	66	10.89
		N/A	501	82.67
13.	Publish the findings of ICMR-STS research?	Yes	12	1.98
		No	87	14.36
		N/A	507	83.66

**Table 6:** Determinants of enthusiasm in research activities in medical colleges. *Strongly agree and Agree were grouped as Group A ("Enthusiastic" about research) **Neutral, Disagree and Strongly disagree were grouped as Group B ("Less interested" in research) ***Indian- INICET and NEET-PG are grouped together since research experience is not needed to get admission in postgraduate courses *****Outside- USMLE and PLAB are grouped together since research experience is needed to get admission in postgraduate courses. USMLE: United States Medical Licensing Examination, NEET-PG: National Eligibility cum Entrance Test (Postgraduate), PLAB: Professional and Linguistic Assessments Board, INICET: Institute of National Importance Combined Entrance Test

	Group A*	Percentage (%)	Group B**	Percentage (%)
India***	136	62.10046	83	219
Outside****	364	95.53806	17	381

**Table 7:** Determinants of enthusiasm in research activities in medical colleges. Interpretation: Enthusiasm in research is very highly determined on the type of postgraduate exam the student is preparing for.

	India	Outside exams	p-value
	n=219	n=381	
Group A (Enthusiastic participants)	62.1	95.53	less than 0.001 (Highly significant)

Table 8 shows the determinants of enthusiasm in research activities in medical colleges. Table 9 shows the determinants of enthusiasm in research activities in medical colleges. Table 10 shows the availability of support for conducting research and related activities to students and graduates.

**Table 8:** Determinants of Enthusiasm in research activities in medical colleges. *Strongly agree and Agree were grouped as Group A ("Enthusiastic" about research) **Neutral, Disagree and Strongly disagree were grouped as Group B ("Less interested" in research)

	Group A*	Percentage (%)	Group B**	Percentage (%)	Total
Private	208	81.6	47	18.43	255
Government	247	81.5	56	18.48	303
Other (excluded due to low values)	48		0		48
					606

**Table 9:** Determinants of Enthusiasm in research activities in medical colleges. Interpretation: Interest in research activities is not determined by the type of colleges - government or private.

	Private	Government	p value
	n=255	n=303	
Group A (Enthusiastic participants)	81.60%	81.50%	0.4879 (Not Significant

**Table 10:** Availability of support for conducting research and related activities to students and graduates. MBBS: Bachelor of Medicine, Bachelor of Surgery

Sr. No.	Help given/sought	Answers	Number (Out of 606)	Percentage (%)
1.	Medical school encouraging students	Strongly Agree	123	20.30
		Agree	177	29.21
		Neutral	171	28.22
		Disagree	90	14.85
		Strongly disagree	45	7.43
		Unanswered	0	0
2.	MBBS batchmates	Strongly Agree	102	16.83
		Agree	207	34.16
		Neutral	225	37.13
		Disagree	57	9.41
		Strongly disagree	15	2.48
		Unanswered	0	0
3.	Were MBBS batchmates helpful?	Strongly Agree	69	11.39
		Agree	138	22.77
		Neutral	192	31.68
		Disagree	69	11.39
		Strongly disagree	18	2.97
		Unanswered	120	19.80
4.	MBBS seniors	Strongly Agree	93	15.35
		Agree	183	30.20
		Neutral	231	38.12
		Disagree	72	11.88
		Strongly disagree	27	4.46
		Unanswered	0	0
5.	Were MBBS seniors helpful?	Strongly Agree	60	9.90
		Agree	141	23.27
		Neutral	165	27.23
		Disagree	54	8.91
		Strongly disagree	33	5.45
		Unanswered	153	25.25
6.	Professors/Faculty	Strongly Agree	159	26.24
		Agree	237	39.11
		Neutral	120	19.80
		Disagree	69	11.39
		Strongly disagree	21	3.47
		Unanswered	0	0
7.	Were Professors/Faculty helpful?	Strongly Agree	96	15.35
		Agree	162	26.73
		Neutral	162	26.73
		Disagree	39	6.44
		Strongly disagree	21	3.47
		Unanswered	102	16.83
8.	Outside Medical college (doctors not in the same college)	Strongly Agree	93	15.35
		Agree	162	26.73
		Neutral	177	29.21
		Disagree	132	21.78
		Strongly disagree	42	6.93
		Unanswered	0	0
9.	Were they helpful?	Strongly Agree	54	9.91
		Agree	117	19.31
		Neutral	219	36.14
		Disagree	39	6.44
		Strongly disagree	15	2.48
		Unanswered	162	26.73
10.	Online research conducting platforms	Strongly Agree	129	21.29
		Agree	171	28.22
		Neutral	165	27.23
		Disagree	102	16.83
		Strongly disagree	39	6.44
		Unanswered	0	0
11.	Were Online platforms helpful?	Strongly Agree	66	10.89
		Agree	153	25.25
		Neutral	168	27.72
		Disagree	33	5.45
		Strongly disagree	15	2.48
		Unanswered	171	28.22

## Discussion

The authors conducted a survey with 606 students from various medical colleges in India to assess their knowledge and passion for research as an important component of their undergraduate education. It is well envisaged that research is an asset for any medical professional to provide the best evidence-based treatment. It enables an individual to gather sufficient data, analyze the information and effectively come to a well-balanced conclusion. In India, many undergraduate students do not value research for a variety of reasons, which the authors set out to investigate. Taking part in enhancing this component is critical to stay up with the rapid speed of new discoveries and the vast amount of medical information being unearthed.

This study comprised 53.47% women and 46.53% men, with 45% of graduates graduating within two years of medical school and a relatively identical percentage of the remaining 55% of MBBS participants (11.3%, 9.9%, 10.87%, 9.41%, and 12.87%). Government and private school participants were in similar proportion. The participants were mostly targeting USMLE (54.95%) followed by NEET-PG/INICET (36.14%) and PLAB/United Kingdom Medical Licensing Assessment (UKMLA) (7.92%). There appears to be no substantial variation in research knowledge among students in government or private medical schools; students writing Indian or international postgraduate admission examinations. Whilst there is no substantial variation in the eagerness to participate in research activities between government and private medical schools, there is a considerable difference between international test-writing students and Indian exam-writing students.

Majority of the participants (78.22%) do consider research and publication important for their overall improvement like those in other countries like Canada [9]. Whilst medical research was presented by their medical school's professors (30.20%), most students (65.68%) first heard about it via their colleagues (batchmates, seniors, and peers from other medical colleges) and even from family (3.96%). The deficit in awareness of research in undergraduate courses is seen as only 39.60% were introduced to the concept of medical research in their first year and there were still participants who got to know about it after they graduated (6.44%). Just 49.5% say their medical school encouraged them to participate in medical research. Just 25.25% of the 82.6% who are interested in research have engaged in any research activity. All of this demonstrates the medical curriculum's inadequate attempts to promote research participation.

When tested on their existing knowledge only approximately 55.2% of the questions were answered correctly with a mean of 2.75±1.32(SD) correct answers per student. Research does not have to be portrayed as a difficult, time-consuming task. Beginning with a fundamental research activity, such as a case report or case series, will help students comprehend and adjust to the process of research publishing. Yet only 7.43% of the 84.65% of participants who were interested were able to publish even a case report/series. It follows a similar pattern for all other types, whether it be original research, a review article, or oral/poster presentations.

The establishment of the ICMR-STS is a significant step forward in terms of medical student awareness and engagement. Yet, it is insufficient to handle the large number of students engaged in research on its own [10-12]. Just 77.23% of our participants were aware of the ICMR-STS programme, and 16.34% applied for it. Nevertheless, just 6.4% completed their study within the term specified, and only 1.98% published their ICMR findings.

With aspirations of still being a part of the research, over half of the participants have reached out for help and guidance [13,14]. They attempted to contact their faculty (65.35%), however just 46.53% were helpful. Just around 34.18% of their classmates and 33.17% of their seniors assisted them. Only 28.22% of those who sought guidance from their colleagues in other medical schools were helpful. Even while 49.51% used online research platforms, only 36.14% benefited from them.

There are various impediments to research involvement throughout a physician's education and training, including time demands both inside and outside of medicine. Apart from a shortage of time, there appear to be other substantial impediments to research engagement throughout medical school, such as the availability of research mentors and a perceived lack of formal education in research technique and critical evaluation [1]. The literature is well aware of the advantages of research exposure for short-term as well as long-term scientific output, and research involvement in medical school is consistently linked to later participation in research projects, including the decision to pursue an academic career. It is essential to have a strong support system in place to encourage research.

Limitations

This study has certain drawbacks. First, the results are based on a self-report survey of medical school students' engagement and attitudes towards research, and independent verification of data was not available. Second, some respondents elected not to answer all of the survey questions. The reach and determination of students to participate in research activity is the main limiting factor regardless of the type and year of medical school. Igniting a passionate interest in medical research in all undergraduate medical students is a tremendous undertaking.

## Conclusions

In the age of information overload, employing an evidence-based approach is a step towards being a well-rounded physician. Medical research should be ardently pursued by all medical personnel starting as early as the first year of undergraduate education. Medical curricula are already overburdened and most of the students are examination oriented and would hate additional burden of research-oriented programs. The medical curriculum should incorporate all aids to assist and avoid the many problems that medical students confront when conducting research. This may be accomplished by including seminars, courses, interactive discussions, and other activities into the medical curriculum while stressing medical research as an essential skill of a medical practitioner. Our goal should be to expand the number of medical researchers as well as research-oriented doctors. Medical research improves best practices by refining clinical guidelines, enhances early screening through effective diagnostic tools, and optimizes healthcare delivery by evaluating and improving systems. It also boosts local healthcare by assessing resources, operations, and outcomes, tailoring solutions to meet specific needs.
